# Embelin-induced MCF-7 breast cancer cell apoptosis and blockade of MCF-7 cells in the G2/M phase via the mitochondrial pathway

**DOI:** 10.3892/ol.2012.1084

**Published:** 2012-12-19

**Authors:** YANG LI, DALEI LI, SHENGGUANG YUAN, ZHENRAN WANG, FANG TANG, RONGRONG NIE, JUN WENG, LINA MA, BO TANG

**Affiliations:** 1 Department of Medical Oncology, Affiliated Hospital of Guilin Medical University, Guilin 541001;; 2 Gamma Knife Center, The Second Hospital of Changchun, Changchun 130062;; 3 Departments of Hepatobiliary Surgery, Affiliated Hospital of Guilin Medical University, Guilin 541001, P.R. China; 4 General Surgery, Affiliated Hospital of Guilin Medical University, Guilin 541001, P.R. China

**Keywords:** embelin, XIAP, breast cancer MCF-7 cell, apoptosis, mitochondria

## Abstract

Embelin is a small molecular inhibitor extracted from *Myrsinaceae* plants that specifically inhibits XIAP, affecting the proliferation and apoptosis of various types of tumor cells. In our previous studies, we have demonstrated that embelin is able to induce the apoptosis of MCF-7 breast cancer cells in a dose-dependent manner. However, its mechanism of action is not yet clear. The purpose of this study was to investigate the involvement of the mitochondrial pathway in embelin-induced apoptosis and the effect of embelin on the cell cycle. Different doses of embelin were added to MCF-7 breast cancer cells and it was found that embelin was able to induce apoptosis of MCF-7 breast cancer cells in a dose- and time-dependent manner. Flow cytometry analysis revealed that embelin caused changes in the MCF-7 cell mitochondrial membrane potential and blocked the cell cycle of MCF-7 cells in the G2/M phase. Moreover, embelin was demonstrated to promote mitochondrial release of cytochrome C via regulation of Bax and Bcl-2, resulting in the activation of caspase-3 and -9, while no significant changes in the level of caspase-8 were observed. The results have demonstrated that embelin-induced apoptosis of MCF-7 breast cancer cells involves the mitochondrial pathway.

## Introduction

Breast cancer is a serious disease that threatens the health of individuals and is the most common malignant tumor in females. It is a major disease affecting females in particular, and its incidence is increasing annually (1–3). The key therapeutic approach for most breast cancer patients is to find an effective antitumor drug to assist surgical treatment.

Embelin is a small molecular inhibitor extracted from *Myrsinaceae* plants. It is a polyphenolic compound that inhibits XIAP by binding to the Smac binding site in the BIR3 domain of XIAP protein molecules (4–6). Previous studies have demonstrated that embelin has anti-inflammatory and anti-oxidative biological effects (7–10). It has been demonstrated that embelin has an extensive antitumor role and is able to restrain the growth of various tumor cells, including those of breast, colon, prostate and pancreatic cancer (11–14). However, the detailed mechanism of the antitumor activity of embelin remains unknown.

This study was designed to investigate the effect of embelin on cell apoptosis and the cell cycle of MCF-7 breast cancer cells *in vitro*, and to explore the embelin-induced cell apoptosis signaling pathway in MCF-7 cells. We have demonstrated that by regulating the Bax and Bcl-2 proteins, embelin induces the release of cytochrome C and activates the caspase family to induce the apoptosis of breast cancer cells.

## Materials and methods

### Cell culture

MCF-7 breast cancer cells were purchased from the American Type Culture Collection (ATCC, Manassas, VA, USA). Following cell passage, cells were inoculated in RPMI-1640 culture medium (Gibco-BRL, Grand Island, NY, USA) containing 10% fetal calf serum (Hyclone Laboratories, Inc., Logan, UT, USA), 100 U/ml penicillin and 100 U/ml streptomycin. The cells were then cultured in an incubator containing 5% CO_2_ and 95% O_2_, at 37°C.

### Cell viability

MCF-7 cells in the logarithmic growth phase were collected and cultured in 96-well culture plates inoculated at a density of 1×10^5^ cells/ml for 24 h. When the cells had grown to adherence, different doses of embelin were administered to various groups with 8 duplicate wells for each concentration. A negative control group without embelin was also created. All cells were incubated in 5% CO_2_ for further culture for 24, 48 and 72 h prior to the color reaction. Subsequently, MTT (5 mg/ml; 20 *μ*l) was added to each well, and cells were cultured in CO_2_ at 37°C in an incubator for 4 h. The culture solution was then diposed of. DMSO (150 *μ*l) was added to each well for room temperature oscillation for 10 min, and the optical density (OD) values (*A*_570nm_) of each well were measured with a microplate reader (Bio-Rad; Hercules, CA, USA)

### Analysis of apoptosis via flow cytometry

A 0.25% trypsin digest was used to collect MCF-7 cells of all groups, and the cell density was adjusted to 1×10^6^ cells/ml. Annexin V-FITC (5 *μ*l) and 5 ml of propidium iodide (PI) were added for 30 min at 4°C to avoid light dyeing, prior to the flow cytometry analysis.

### Analysis of cell cycle by flow cytometry

MCF-7 cells of all groups were digested and collected using 0.25% trypsin, and then washed with PBS solution. Cells were fixed at 4°C with 75% cold ethanol overnight and washed with PBS solution. The cell density was adjusted to 1×10^6^ cells/ml and the final volume was 100 *μ*l. DNAStain comprehensive dye liquor (500 ml; Sigma, St. Louis, MO, USA) was added for storage at room temperature in a dark place for 30 min prior to testing with flow cytometry. The DNAStain contained RNase, PI and Triton X-100 at end concentrations of 50mg/l, 100 mg/l and 1 ml/l, respectively.

### Flow cytometric analysis of mitochondrial membrane potential

The cell density was adjusted to 1×10^6^ cells/ml and JC-1 dye with an end concentration of 10 mg/ml was added. This was then mixed and cultured for 30 min at 37°C in the dark. Analysis with flow cytometry followed. FL1-H indicated a green fluorescence intensity and FL2-H was a red fluorescence intensity. Quantitative analysis was conducted using the CellQuest analysis software.

### Western blot analysis

MCF-7 cells of all groups were collected using a trypsin digest and 2 ml of lysis solution (50 mM Tris-HCl, 137 mM NaCl, 10% glycerin, 100 mM sodium vanadate, 1 mM PMSF, 10 mg/ml aprotinin, 10 mg/ml leupeptin, 1% NP-40 and 5 mM cocktail; pH 7.4) was added for cell lysis to obtain the proteins. Following the BCA assay, proteins were loaded onto a gel and separated by SDS-PAGE. The proteins were then transferred to a PVDF membrane using a semi-dry method and sealed with 5% skimmed mild powder at 4°C overnight. The membrane was washed with Tris-buffered saline Tween-20 (TBST) and the first antibody was added at 37°C for hybridization for 1 h, prior to bleaching with TBST. The second antibody was then added at 37°C for hybridization for 1 h prior to bleaching with TBST and a color reaction for 5 min with autoradiography. Quantity One software was used for OD value analysis and measurements.

### Statistical analysis

The SPSS software, version 16.0 was used for assessing dependence and for variance analysis. Values are presented as mean ± standard deviation. P<0.05 was considered to indicate a statistically significant difference between groups.

## Results

### Embelin induces inhibition of MCF-7 breast cancer cell growth

**Figure 1:**
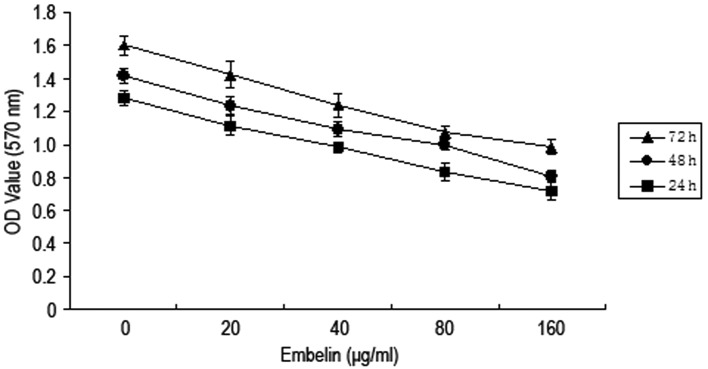
Embelin-induced inhibition of MCF-7 breast cancer cell growth. Cell viability was examined with the MTT method following the addition of different concentrations of embelin (0, 20, 40, 80 and 160 *μ*g/ml) to MCF-7 cells for 24, 48 and 72 h. The results are representative of eight independent experiments. OD, optical density.

Different concentrations of embelin (0, 20, 40, 80 and 160 *μ*g/ml) were added to MCF-7 breast cancer cells for 24, 48 and 72 h. The MTT method was then employed to determine the cell activity (Fig. 1). The results demonstrated that A_570_ values of breast cancer MCF-7 cells gradually decreased when the concentration of embelin was increased, and the A_570_ value decreased most when the concentration of embelin was 80 *μ*g/ml. This indicates that embelin has an inhibitory effect on the growth of MCF-7 breast cancer cells.

### Embelin induces apoptosis of MCF-7 breast cancer cells

**Figure 2:**
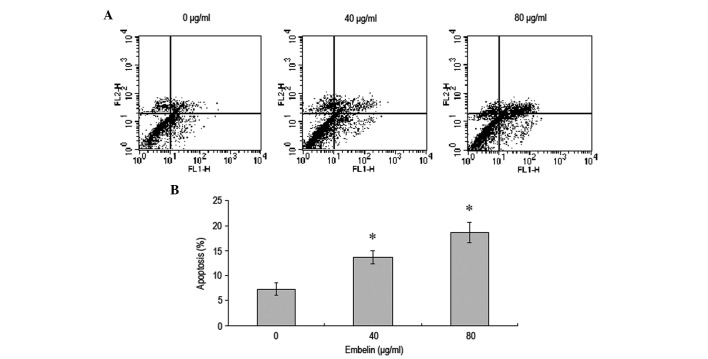
Embelin induced MCF-7 breast cancer cell apoptosis. (A) Annexin V/propidium iodide (PI) dye was used to determine the rate of apoptosis following treatment of MCF-7 cells with different concentrations of embelin (0, 40 and 80 *μ*g/ml) for 48 h. (B) The histogram shows the apopotosis rate (%) of MCF-7 cells. ^*^P<0.05 vs. control. The data are representative of three independent experiments.

MCF-7 breast cancer cells were treated with different concentrations of embelin for 48 h and flow cytometry was used to investigate the effect on the rate of apoptosis (Fig. 2). Our results demonstrated that, with an increase in the concentration of embelin, the rate of MCF-7 breast cancer cell apoptosis significantly increased in a dose-dependent manner.

### Embelin induces apoptosis of MCF-7 breast cancer cells via the mitochondrial pathway

**Figure 3:**
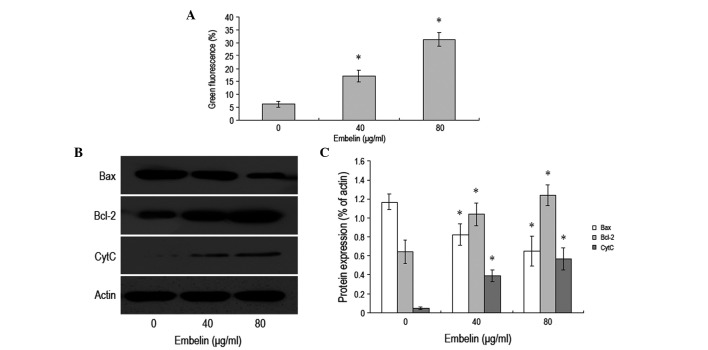
Embelin induced MCF-7 breast cancer cell apoptosis via the mitochondrial pathway, following treatment of MCF-7 cells with different concentrations of embelin (0, 40 and 80 *μ*g/ml) for 48 h. (A) JC-1 dye flow cytometry was used to analyze the change in the mitochondrial membrane potential. (B) Western blot analysis was used to determine the expression levels of Bax, Bcl-2 and cytochrome C proteins inside the cytoplasm. (C) The western blot analysis results were further analyzed using Gel-Pro Analyzer 4.0 software. ^*^P<0.05 vs. control. Results are representative of three independent experiments.

JC-1 coloration was used to examine the changes in the mitochondrial membrane potential. The results demonstrated a gradual decrease in the mitochondrial membrane potential along with an increase in the concentration of embelin. Additionally, the change in the mitochondrial membrane potential was observed prior to apoptosis (Fig. 3A). Western blot analysis was employed to examine the levels of Bax, Bcl-2 and cytochrome C inside the cytoplasm. It was revelaed that when the concentration of embelin was increased, the level of Bax protein gradually decreased, while the level of cytochrome C increased (Fig. 3B and C). Furthermore, the level of Bcl-2 protein was observed to increase when the concentration of embelin was increased. Our data demonstrated that embelin is able to change the mitochondrial membrane potential to promote a change in the levels of Bax and Bcl-2 as well as the release of cytochrome C, which results in the apoptosis of MCF-7 breast cancer cells.

### Effect of embelin on the expression levels of apoptosis-related proteins in MCF-7 breast cancer cells

**Figure 4:**
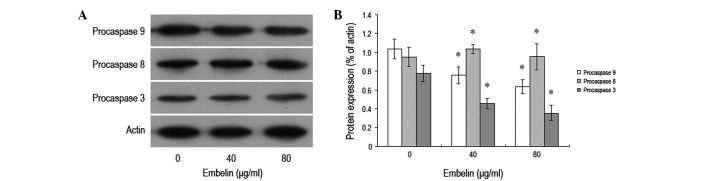
Effect of embelin on the expression levels of MCF-7 breast cancer cell apoptosis-related proteins, including procaspase-3, -8 and -9, 48 h following the treatment of MCF-7 breast cancer cells with different concentrations of embelin (0, 40 and 80 *μ*g/ml). (A) Western blot analysis was used to analyze the expression of procaspase-3, -8 and -9 proteins. (B) The western blot analysis results were further analyzed using Gel-Pro Analyzer 4.0 software. ^*^P<0.05 vs. control. Results are representative of three independent experiments.

In order to study the effect of embelin on the expression levels of MCF-7 breast cancer cell apoptosis-related proteins, different concentrations of embelin were administered to MCF-7 cells for 48 h, and western blot analysis was conducted to investigate the expression levels of procaspase-3, -8 and -9 proteins (Fig. 4). It was found that following treatment with different concentrations of embelin, the expression levels of MCF-7 breast cancer cell procaspase-3 and -9 proteins signficantly decreased, while no significant differences were observed in the expression level of procaspase-8 protein. Our data demonstrated that embelin-induced MCF-7 apoptosis may occurr through the mitochondria-mediated caspase-3 and -9 pathways.

### Embelin-induces MCF-7 breast cancer cell cycle blockade in G2/M phase

**Figure. 5:**
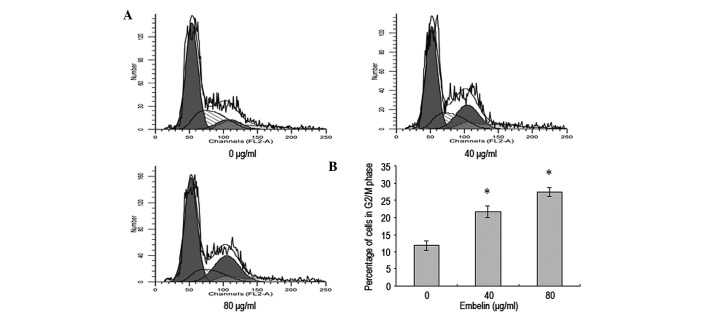
Effect of embelin on the cell cycle of MCF-7 breast cancer cells, following treatment of the cells with different concentrations of embelin (0, 40 and 80 *μ*g/ml) for 48 h. (A) Flow cytometry was used to analyze the cell cycle. (B) The histogram shows the percentage of cells in G2/M phase of the cell cycle. ^*^P<0.05 vs. control. The data are representative of three independent experiments.

Flow cytometry was conducted to investigate whether embelin affected the cell cycle of MCF-7 breast cells. The results revealed that 48 h following the addition of different concentrations of embelin to MCF-7 cells, a cell cycle blockade was observed in the G2/M phase compared with the control group (Fig. 5). This suggests that embelin is able to increase the percentage of MCF-7 cells in the G2/M phase to decrease the proliferation of breast cancer cells.

## Discussion

Kerr *et al*(15) first proposed the concept of apoptosis. Apoptosis is ubiquitous in the majority of tumor cells, and is important in the genesis and progression of tumors (16). Previous studies have demonstrated that antitumor drugs typically inhibit tumors by inducing apoptosis of sensitive tumor cells. Therefore, the intervention in apoptosis to treat tumors has become a new target in the search for antitumor drugs and a new development direction in present tumor pharmacology.

Breast cancer is a potentially life-threatening malignant tumor; it is important to study the disease to find effective anti-tumor drugs. Antitumor drugs that originate from plants have benefits including an extensive variety, low toxicity and few side-effects and adverse reactions. Therefore, highly effective antitumor drugs from plants are being explored. Embelin is a small molecular inhibitor with specific inhibition of XIAP that affects the proliferation and apoptosis of various tumor cells. Certain studies have demonstrated that embelin inhibits the proliferation of various tumor cells; significant effects have been observed in breast cancer and other solid tumor cells (17). The results of the present study are concordant with these findings. We demonstrated that when breast cancer cells had been treated with different concentrations of embelin for 48 h, the rate of cell apoptosis increased in a dose-dependent manner, indicating that embelin is able to induce breast cancer cell apoptosis as opposed to directly causing cell death. Moreover, embelin has the potent effect of restraining the cell cycle transition of breast cancer cells to blockade the cell cycle in G2/M phase, therefore altering the progression of the cell cycle to induce apoptosis.

There are various signaling pathways of apoptosis within an organism, of which the mitochondrial pathway is one of the most important. Bcl-2 family proteins are key regulatory factors of the mitochondrial pathway (18–20). We demonstrated that that when breast cancer cells had been treated with different doses of embelin for 48 h, Bax and Bcl-2 migrated, the mitochondrial membrane potential increase expression of Bax, while Bcl-2 expression decreased and cytochrome C was released. These results indicated that the induction of breast cancer cell apoptosis by embelin was closely associated with the mitochondrial pathway. As demonstrated in previous studies, with the stimulation of pro-apoptosis factors, the Bax protein migrated from the cytoplasm to the outer mitochondrial membrane, changing the permeability of the outer mitochondrial membrane to promote the mitochondrial release of cytochrome C (21). Moreover, Bcl-2 protein is able to stabilize the mitochondrial permeability transition pore (mPTP) and maintain the normal functioning of the pore. In the present study, we demonstrated that with embelin treatment, the Bcl-2 protein level inside the cytoplasm gradually decreased while the release of cytochrome C increased in a stepwise manner. This indicates that embelin induces the activity of Bcl-2, causing the mPTP to open irreversibly, which further changes the permeability of the mitochondrial membrane and promotes the release of cytochrome C. The combination of these factors results in apoptosis.

The caspase family activates apoptosis-related protease when apoptosis occurs (22,23), and a series of subsequent biological effects occur in turn. Therefore, activation of the caspase family is important in the process of apoptosis. We analyzed the changes in procaspase-3, -8 and -9 proteins following treatment of breast cancer cells with embelin. A significant decrease in procaspase-3 and -9 expression was observed when breast cancer apoptosis occured, but no changes in the level of procaspase 8 expression were identified. The effect of releasing cytochrome C from the mitochondria to activate caspase-3 and -9 is an important step of the apoptotic pathway (24,25). These results suggest that embelin-induced apoptosis of breast cancer cells is realized via the endogenous mitochondrial pathway as opposed to via the exogenous death receptor pathway.

In summary, our study has demonstrated that embelin releases cytochrome C and activates the caspase family to result in the induction of breast cancer apoptosis through regulation of the action of the Bcl-2/Bax family in the mitochondrial pathway. Embelin may offer important contributions for the development of a novel drug to prevent and cure breast cancer in the future.

## References

[1] McPherson K, Steel CM, Dixon JM (2000). ABC of breast diseases. Breast cancer-epidemiology, risk factors, and genetics. BMJ.

[2] Benson JR, Jatoi I (2012). The global breast cancer burden. Future Oncol.

[3] Anderson WF, Katki HA, Rosenberg PS (2011). Incidence of breast cancer in the United States: current and future trends. J Natl Cancer Inst.

[4] Reuter S, Prasad S, Phromnoi K, Kannappan R, Yadav VR, Aggarwal BB (2010). Embelin suppresses osteoclastogenesis induced by receptor activator of NF-κB ligand and tumor cells in vitro through inhibition of the NF-κB cell signaling pathway. Mol Cancer Res.

[5] Hu R, Zhu K, Li Y, Yao K, Zhang R, Wang H, Yang W, Liu Z (2011). Embelin induces apoptosis through down-regulation of XIAP in human leukemia cells. Med Oncol.

[6] Nikolovska-Coleska Z, Xu L, Hu Z, Tomita Y, Li P, Roller PP, Wang R, Fang X, Guo R, Zhang M, Lippman ME, Yang D, Wang S (2004). Discovery of embelin as a cell-permeable, small-molecular weight inhibitor of XIAP through structure-based computational screening of a traditional herbal medicine three-dimensional structure database. J Med Chem.

[7] Joshi R, Kamat JP, Mukherjee T (2007). Free radical scavenging reactions and antioxidant activity of embelin: biochemical and pulse radiolytic studies. Chem Biol Interact.

[8] Sreepriya M, Bali G (2006). Effects of administration of Embelin and Curcumin on lipid peroxidation, hepatic glutathione antioxidant defense and hematopoietic system during N-nitrosodiethylamine/Phenobarbital-induced hepatocarcinogenesis in Wistar rats. Mol Cell Biochem.

[9] Mahendran S, Badami S, Ravi S, Thippeswamy BS, Veerapur VP (2011). Synthesis and evaluation of analgesic and anti- inflammatory activities of most active free radical scavenging derivatives of embelin-A structure-activity relationship. Chem Pharm Bull.

[10] Thippeswamy BS, Mahendran S, Biradar MI, Raj P, Srivastava K, Badami S, Veerapur VP (2011). Protective effect of embelin against acetic acid induced ulcerative colitis in rats. Eur J Pharmacol.

[11] Danquah M, Li F, Duke CB, Miller DD, Mahato RI (2009). Micellar delivery of bicalutamide and embelin for treating prostate cancer. Pharm Res.

[12] Dai Y, Qiao L, Chan KW, Yang M, Ye J, Ma J, Zou B, Gu Q, Wang J, Pang R, Lan HY, Wong BC (2009). Peroxisome proliferator-activated receptor-gamma contributes to the inhibitory effects of Embelin on colon carcinogenesis. Cancer Res.

[13] Aird KM, Ding X, Baras A, Wei J, Morse MA, Clay T, Lyerly HK, Devi GR (2008). Trastuzumab signaling in ErbB2-overexpressing inflammatory breast cancer correlates with X-linked inhibitor of apoptosis protein expression. Mol Cancer Ther.

[14] Mori T, Doi R, Kida A, Nagai K, Kami K, Ito D, Toyoda E, Kawaguchi Y, Uemoto S (2007). Effect of the XIAP inhibitor Embelin on TRAIL-induced apoptosis of pancreatic cancer cells. J Surg Res.

[15] Kerr JF, Wyllie AH, Currie AR (1972). Apoptosis: a basic biological phenomenon with wide-ranging implications in tissue kinetics. Br J Cancer.

[16] Chiarugi P, Giannoni E (2008). Anoikis: a necessary death program for anchorage-dependent cells. Biochem Pharmacol.

[17] Allensworth JL, Aird KM, Aldrich AJ, Batinic-Haberle I, Devi GR (2012). XIAP inhibition and generation of reactive oxygen species enhances TRAIL sensitivity in inflammatory breast cancer cells. Mol Cancer Ther.

[18] Tang B, Zhang Y, Liang R, Yuan P, Du J, Wang H, Wang L (2011). Activation of the δ-opioid receptor inhibits serum deprivation-induced apoptosis of human liver cells via the activation of PKC and the mitochondrial pathway. Int J Mol Med.

[19] Mattson MP, Kroemer G (2003). Mitochondria in cell death: novel targets for neuroprotection and cardioprotection. Trends Mol Med.

[20] Burlacu A (2003). Regulation of apoptosis by Bcl-2 family proteins. J Cell Mol Med.

[21] Saito M, Korsmeyer SJ, Schlesinger PH (2000). BAX-dependent transport of cytochrome c reconstituted in pure liposomes. Nat Cell Biol.

[22] Nicholson DW, Ali A, Thornberry NA, Vaillancourt JP, Ding CK, Gallant M, Gareau Y, Griffin PR, Labelle M, Lazebnik YA (1995). Identification and inhibition of the ICE/CED-3 protease necessary for mammalian apoptosis. Nature.

[23] Hyman BT, Yuan J (2012). Apoptotic and non-apoptotic roles of caspases in neuronal physiology and pathophysiology. Nat Rev Neurosci.

[24] Riedl SJ, Shi Y (2004). Molecular mechanisms of caspase regulation during apoptosis. Nat Rev Mol Cell Biol.

[25] Shi LG, Zhang GP, Jin HM (2006). Inhibition of microvascular endothelial cell apoptosis by angiopoietin-1 and the involvement of cytochrome C. Chin Med J (Engl).

